# Variability in DNA Methylation and Generational Plasticity in the Lombardy Poplar, a Single Genotype Worldwide Distributed Since the Eighteenth Century

**DOI:** 10.3389/fpls.2018.01635

**Published:** 2018-11-13

**Authors:** An Vanden Broeck, Karen Cox, Rein Brys, Stefano Castiglione, Angela Cicatelli, Francesco Guarino, Berthold Heinze, Marijke Steenackers, Kristine Vander Mijnsbrugge

**Affiliations:** ^1^Research Institute for Nature and Forest (INBO), Geraardsbergen, Belgium; ^2^Department of Chemistry and Biology A. Zambelli, University of Salerno, Fisciano, Italy; ^3^Department of Forest Genetics, Austrian Federal Research Centre for Forests (BFW), Vienna, Austria

**Keywords:** bud phenology, DNA methylation, ecological epigenetics, epigenetic variation, Lombardy poplar, *Populus nigra*, transgenerational plasticity, vegetative propagation

## Abstract

In the absence of genetic diversity, plants rely on the capacity of phenotypic plasticity to cope with shifts in environmental conditions. Understanding the mechanisms behind phenotypic plasticity and how local phenotypic adjustments are transferred to clonal offspring, will provide insight into its ecological and evolutionary significance. Epigenetic changes have recently been proposed to play a crucial role in rapid environmental adaptation. While the contribution of epigenetic changes to phenotypic plasticity has been extensively studied in sexual reproducing model organisms, little work has been done on vegetative generations of asexual reproducing plant species. We studied the variability of DNA methylation and bud set phenology of the Lombardy poplar (*Populus nigra* cv. *Italica* Duroi), a cultivated tree representing a single genotype worldwide distributed since the eighteenth century. Bud set observations and CpG methyl polymorphisms were studied on vegetative offspring resulting from cuttings grown for one season in a common glasshouse environment. The cuttings were collected from 60 adult Lombardy poplars growing in different environments. The physiological condition of the cuttings was determined by measuring weight and nutrient condition. Methylation sensitive amplified polymorphisms were used to obtain global patterns of DNA methylation. Using logistic regression models, we investigated correlations among epigenotype, bud phenology, and the climate at the home site of the donor trees, while accounting for physiological effects. We found significant epigenetic variation as well as significant variation in bud phenology, in the absence of genetic variation. Remarkably, phenology of bud set observed at the end of the growing season in the common environment was significantly correlated with climate variables at the home site of the mother trees, specifically the average temperature of January and monthly potential evapotranspiration. Although we could not directly detect significant effects of epigenetic variation on phenology, our results suggest that, in the Lombardy poplar, epigenetic marks contribute to the variation of phenotypic response that can be transferred onto asexually reproduced offspring resulting in locally adapted ecotypes. This contributes to the growing evidence that epigenetic-based transgenerational inheritance might be relevant for adaptation and evolution in contrasting or rapidly changing environments.

## Introduction

In the absence of genetic diversity, plants rely on the capacity of phenotypic plasticity to cope with shifts in environmental conditions (Castonguay and Angers, 2012). Since many tree and shrub species reproduce asexually, resulting in new individuals (i.e., offspring) that are genetically identical to their parents, it is generally recognized that phenotypic plasticity is a favorable feature to respond to changing environmental conditions. For example, in the field of the domestication and breeding of tree species, phenotypic plasticity has been frequently reported as a camouflaging effect on the breeding value of the genotype (e.g., Houtzagers, 1937; Stearns, 1989; Farmer, 1996). However, surprisingly little is known about the mechanisms behind transgenerational plasticity, especially on how phenotypic adjustments to local conditions are passed onto vegetative offspring, the so called transgenerational phenotypic plasticity (Latzel and Klimešová, 2010; Rohde et al., 2011; Münzbergová and Hadincová, 2017).

Although the processes behind transgenerational plastic effects are not yet perfectly understood, it is generally believed that epigenetic inheritance is one of the most important drivers (e.g., Latzel and Klimešová, 2010; Verhoeven et al., 2010; Richards et al., 2017). DNA methylation, the addition of a methyl group to one of the four bases in the DNA molecule (usually cytosine), is recognized as one of the prime epigenetic mechanisms to correlate with gene expression. Moreover, methyl polymorphisms at CpG sites (cytosine-phosphate-guanine sites where a cytosine is directly followed by a guanine in the DNA sequence) have recently been proposed to a play a crucial role in rapid environmental adaptation (Huang et al., 2017) and may provide asexual organisms with additional sources of variation to cope with contrasting or shifting environmental conditions (e.g., Castonguay and Angers, 2012; Richards et al., 2012; Verhoeven and Preite, 2014). Recently, some studies have indeed shown that epigenetic effects can result in novel phenotypes without any variation in the DNA sequence (Cubas et al., 1999), and that epigenetic states may persist after the initiating factor causing the epigenetic effects disappeared (e.g., Verhoeven et al., 2010; Xie et al., 2015; Xu et al., 2016).

Although most studies on epigenetic inheritance in plants have been done in controlled settings and on sexual model organisms such as Arabidopsis (e.g., Zhang et al., 2013; Cortijo et al., 2014; Dubin et al., 2015), some studies also recently documented the occurrence of epigenetic variation in asexually reproducing plant populations (Richards et al., 2012; Preite et al., 2015; Spens and Douhovnikoff, 2016). Nonetheless, insights into the epigenetic stability over generations and its adaptive significance under real environmental conditions remain, largely unknown (Richards et al., 2017).

Here, we report on the variation in DNA methylation and transgenerational phenotypic variation of the Lombardy poplar (*Populus nigra* cv. *Italica* Duroi), a cultivated variety of *P. nigra* L. that is distributed worldwide since the beginning of the eighteenth century. This clonal variety likely originated between 1700 and 1720 (Elwes and Henry, 1913; Henry, 1914) from one single male mutant tree of *P. nigra* located in central Asia from where it was spread to Europe and other continents (Zsuffa, 1974). In the mid-eighteenth century, the Lombardy poplar was spread by cuttings worldwide from Italy, reaching France in 1749, England in 1758, and North America in 1784 (Wood, 1994). It has been widely introduced for use as windbreaks, screens, avenue trees, and landscape plantings all over the temperate regions of the world (in Europe, North and South America, South Africa, Australia, New Zealand, and China) even in subtropical environments where it appears to perform poorly (CABI, 2017). Its clonal origin in combination with its widespread distribution in space and time, makes the Lombardy poplar an excellent study system to investigate how long-lived plant species with a prevailing vegetative reproduction can cope with widely contrasting environmental conditions, without variation at the genetic level. The Lombardy poplar can be easily and inexpensively propagated by cuttings and vegetative propagation is the only way to conserve the typical columnar tree habit and the unusual vertical branching structure. As a result, most Lombardy poplars originate from artificial vegetative reproduction performed by humans, with plant material that has been grown locally for centuries. It can thus be expected, that the large-scale geographic, but artificial expansion of this cultivar may have resulted in the accumulation of lineage-specific, selectively neutral spontaneous epimutations, and in environmental-directed epigenetic effects that are potentially heritable and may have generated different local phenotypes.

In this work, we used Methylation-Sensitive Amplified Fragment Length Polymorphisms (MS-AFLPs) on cuttings grown in a common environment and collected from 60 adult Lombardy poplars representing a single genotype and located in different climates along a north-south distribution of ca. 2120 km (15.2° latitude) and across an east-west distribution of ca. 1700 km (30.1° longitude). We also studied potential transgenerational effects on bud set as a cornerstone of the seasonal growth cycle (Rohde et al., 2011) on ramets collected on 65 Lombardy poplars and grown in the common environment. We considered the adult Lombardy poplars growing in the different environments as the F_0_-generation, and their vegetative offspring (cuttings) grown in the common glasshouse environment as the F_1_-generation. Specifically, the aims of this study are to test whether; (i) there is significant natural variation in DNA methylation among the widely distributed Lombardy poplar (F_0_) that can persists in clonal offspring (F_1_) in a common glasshouse environment, (ii) the epigenetic differentiation is associated with the maternal growing environment, (iii) there is adaptive phenotypic variation among the Lombardy poplars in terms of bud set, that can persist among vegetatively reproduced F_1_-offspring grown in a common environment, and (iv) the potential variation in bud set is related with the climate of the sampling origin.

## Materials and methods

### Sampling and climate data collection

Dormant twigs of 94 adult putative Lombardy poplar trees (hereafter called; donor trees, F_0_-generation) were collected during the winter of 2016–2017 at in total 37 locations (hereafter called; home sites) in Europe and Asia (Supplementary Table 1). After phytosanitary inspection, the twigs were shipped by express mail to the Research Institute for Nature and Forest located in Geraardsbergen, Belgium (lat. 50,77635°, lon. 3,881007°) and upon arrival stored in the fridge at 4°C until the greenhouse experiment was established. We used publically available global climate data sets to characterize the home environment of each donor tree. Climate variables (Table 1) were calculated for the period 1965–2015 with the R package RFc version 0.1-2. (Grechka et al., 2016).

**Table 1:** Geography and climate data for Lombardy poplar accessions.

**Climate variable**	**Range**	
	**Min**	**Max**
Latitude (degree)	40.752	55.890
Longitude (degree)	−4.593	25.457
Average January Temp (°C)^a^	−1.400	8.024
Average March Temp (°C)	3.87	10.83
Average July Temp (°C)	14.93	26.20
Average precipitation rate (mm/month)^a^	23.274	101.065
Frost days frequency (days per year)^a^	8.497	26.436
Potential evapotranspiration (mm/month)^b^	22.746	43.879

*Variables derived from 1965 to 2015 with the R package RFc version 0.1-2. (Grechka et al., 2016)*.

a *From the climate data-set CRU TS 2.0*.

b *From Climate Malmstrom Air Force Base*.

### Greenhouse experiment

A greenhouse experiment was set up on 9 and 10 March 2017. Only plant material of good quality (1-year old, fresh shoots) of the collected donor trees was included in the experiment. The collected shoots were divided into cuttings of 22 cm in length. Up to 14 cuttings (mean: 12.8, range: 4–14) per donor tree were planted in trays to a depth of about 19 cm after recording the weight of each individual cutting, resulting in a total of 1133 planted cuttings (further called: ramets, F_1_-generation). The trays consisted of 7 × 4 individual cells and were filled with potting soil (50% white peat / 50% black peat, 0.12% nitrogen, 0.14% phosphorous, 0.24% potassium). Ramets were grouped per donor tree within a tray (generally half a tray per tree), and donor trees were randomly distributed among trays. The trays were placed together under similar light and temperature conditions in the greenhouse and were regularly watered. No fertilizers or other soil supplements were provided during the experiment. On 10 May 2017, a fully expanded, fresh leaf was collected for DNA-analysis from the top of a single ramet per donor tree, except for two donor trees (one from Germany (code: GEB1) and one from Spain (code: SPC1) of which leaves were not yet fully unfolded and collected a few days later. For 14 ramets, a second leaf was collected to serve as a replicate. Sampled leaves were dried in silica gel. A list of the Lombardy poplar accessions is given in the additional Supplementary Table 1.

### Nutrient condition of the ramets

Beside epigenetic mechanisms, other, maternally inherited factors may affect bud phenology like the ramets' nutrient condition (e.g., Marchi et al., 2005) which, in turn, relates to the topsoil mineral condition at the site of the donor tree (Cools et al., 2014). For each sampled donor tree, the mineral nutrition condition was determined by measuring total carbon (C) and total nitrogen (N). Foliage samples (5 to 10 leaves, in total) were collected on 18 May 2017 from one to three ramets per donor tree. They were dried in an oven at 40°C for 1 week and pulverized with a blender. For each of the sampled donor trees a homogenized subsample was analyzed. The total N and total C content was determined using a C/N analyzer (Skalar, FormacsHT, Breda, The Netherlands) and expressed per unit of dry biomass (g kg^−1^).

### DNA extraction

Total genomic DNA was extracted from the sampled leaves with the Qiagen Plant DNA kit (Hilden, Germany). The integrity of the DNA was assessed on 1.5% agarose gels, and DNA quantification was performed with Quant-iT™ PicoGreen® dsDNA Assay Kit (Thermo Fisher Scientific, Massachusetts, USA) using a Synergy HT plate reader (BioTek, Vermont, USA).

### SSR analysis

Nuclear microsatellite polymorphisms (SSR) were used to determine the multilocus genotype of the donor trees propagated in the greenhouse experiment. We selected 11 SSRs that were found useful for the identification of *P. nigra* clones in former studies (van der Schoot et al., 2000; Smulders et al., 2001; Liesebach et al., 2010). PCR products were run on an ABI 3500 analyzer with the GeneScan-600 LIZ size standard and analyzed using GeneMapper 4.1 (Thermo Fisher Scientific). The 14 replicated samples were used to calculate the genotyping error rate, calculated as 100 × (number of discordant scores in two independent analyses)/(number of scored markers × number of individuals analyzed). Details on SSRs and PCR-conditions are given in the additional Supplementary Table 2.

### MS-AFLP analysis

The Methylation Sensitive Amplified Length Polymorphism Analysis (MS-AFLP) was performed on vegetative offspring of the donor trees identified as Lombardy poplar based on the results of the SSR-analysis. The MS-AFLP method was adapted from Guarino et al. (2015) using the enzyme combinations *Eco*RI—*Hpa*II and *Eco*RI—*Msp*I. *Hpa*II and *Msp*I cut DNA sequences at the same tetra-nucleotide motif (5′-CCGG-3′), but have different sensitivities to cytosine methylation at the restriction site. This allows the determination of the CpG-methylation status of anonymous regions of the genome. The two MS-AFLP profiles for every sample were compared to identify polymorphic epigenetic loci. We initially tested 32 primer combinations on a subset of 16 samples. Of these primer combinations, seven were selected based on the quality and the reproducibility of amplified bands and the presence of polymorphisms (Table 2). Fourteen samples were replicated, starting from a second leaf sample and two different DNA extractions to assess the reproducibility of the analysis. PCR amplicons were fluorescently labeled with one of two dyes: NED or VIC, and were run in simplex on an ABI 3500 analyzer with the GeneScan-600 LIZ size standard (Thermo Fisher Scientific). We used GeneMapper v4.1 (Thermo Fisher Scientific) for the sizing of the DNA fragments. The quality of the electropherograms was visually checked in GeneMapper and electropherograms of low quality (e.g., weaker profiles with unreliable and/or low peak intensities) were removed before importing peak data into RawGeno version 2.0-1 (Arrigo et al., 2009), an R package for automatic scoring of AFLP datasets. Only fragments ≥150 bp in size were considered to reduce the potential impact of size homoplasy (Vekemans et al., 2002). DNA fragment profiles were processed per *Eco*RI/*Hpa*II—*Msp*I primer combination pairs and scored in RawGeno using the scoring parameters given in the additional Supplementary Table 3. Singletons were removed from the data. The genotyping error rate was calculated per *Eco*RI/*Hpa*II—*Msp*I primer combination pairs in RawGeno according to Bonin et al. (2004). After removing samples with missing data, the binary data of each of the seven primer combinations were combined resulting in a data matrix of complete *Eco*RI/*Hpa*II and *Eco*RI/*Msp*I fragment profiles for vegetative offspring (F_1_-generation) of 60 donor trees (the 14 replicates excluded) and 226 loci. We used the methylation scoring approach described in Herrera and Bazaga (2010) to transform this data matrix into a binary data matrix representing the epigenetic diversity. The absence of fragments of both *Hpa*II and *Msp*I cuts (condition 2 in Herrera and Bazaga, 2010) represents either methylation of both (internal and external) cytosines or absence of the digestion site via mutation (Schulz et al., 2013). We scored the absence of fragments of both *Hpa*II and *Msp*I cuts as uninformative (missing data) to account for somatic mutations. Only loci exceeding a specific methylation threshold were scored. This threshold was specific for each primer combination (Table 2) and set equal to the expected per-individual probability of obtaining a mismatch of *Hpa*II and *Msp*I scores owing to technical and/or scoring errors (see Herrera and Bazaga, 2010). We use the term “epigenotypes” to refer to the epigenetically polymorphic CCGG sites resulting from the MS-AFLP-analysis. The R package msap version 1.1.8 (Pérez-Figueroa, 2013) was used to transform the absence and presence of fragments of both *Hpa*II and *Msp*I cuts into epigenotypes. The resulting binary data matrix of polymorphic methylation-sensitive markers was used for analyses of epigenetic data in GenAlEx version 6.4 (Peakall and Smouse, 2012). We identified shared epigenotypes among individuals (considering missing data when finding matches), estimated haplotype diversity, computed the Shannon's Diversity Index and performed a principal coordinate analysis (PCoA) to determine and visualize the epigenetic variation and structure of the analyzed ramets. We then performed an analysis of molecular variance (AMOVA) with samples grouped per country to determine the epigenetic differences among Lombardy poplar ramets sampled in different countries calculated as mean pairwise Φ_ST_ distances. In two countries (Spain and Bosnia Herzegovina), only one tree each was sampled, these were removed prior to the AMOVA approach. The probability for significance of Φ_ST_ was based on 999 permutations across the full data set (Michalakis and Excoffier, 1996). Mantel test analysis (Hutchison and Templeton, 1999) was used to estimate the correlation between the Euclidean epigenetic distance matrix generated by GenAlEx and the geographic distance matrix of sampled trees (km). The significance of the Mantel test was assigned by random permutations tests (based on 999 replicates).

**Table 2:** Characteristics of the primer combinations used in the MS-AFLP analysis of 60 Lombardy poplars grown in a common greenhouse environment.

	**Primer combination**	**Total MS-AFLP markers in the size range 150–600 bp**	**Scoring error rate^a^**	**Methylation-susceptible markers^b^**	
				**N**	**N Polymorphic (%)**
1	*Eco*RI + ACC/*Hpa*II-*Msp*I + TAC	29	0.029	16	9 (56.25%)
2	*Eco*RI + ACC/*Hpa*II-*Msp*I + TAG	22	0.000	21	16 (76.19%)
3	*Eco*RI + AGC/*Hpa*II-*Msp*I + TCC	25	0.024	13	13 (100%)
4	*Eco*RI + AGC/*Hpa*II-*Msp*I + TCT	28	0.033	10	3 (30%)
5	*Eco*RI + AGC/*Hpa*II-*Msp*I + TCG	20	0.038	9	4 (44.44%)
6	*Eco*RI + AGC/*Hpa*II-*Msp*I + TAA	51	0.044	10	10 (100%)
7	*Eco*RI + ACT/*Hpa*II-*Msp*I + TAG	41	0.028	15	10 (66.67%)
	All combined	216	0.028	94	65 (67.65%)

a *Calculated by RawGeno according to Bonin et al. (2004) and per EcoRI/HpaII – MspI primer pairs on the DNA fingerprinting profiles from the 14 replicated samples*.

b *N, number of methylation-susceptible markers. A methylation-susceptible marker was considered polymorphic when both methylated and non-methylated states occurred in the total sample of 60 individuals*.

We applied simple logistic regression models to investigate whether the variation in DNA-methylation observed at a particular epilocus, depends on the environmental variables recorded at the location of the donor trees. For this analysis, we ignored possible somatic mutations and scored fragment absence as unmethylated (score: “0”). We included as exploratory variables the average temperature (°C) of the coldest (January) and warmest (July) month in the year and of March (temperature in early spring), the average monthly precipitation rate (mm month^−1^), the average number of frost days per year and the average monthly potential evapotranspiration rate (PET) (mm month^−1^). We also tested if the epigenetic variation is related to the topsoil nutrient availability (carbon-nitrogen ratio or CN) on the location of the donor tree. The analyses were performed in R using the generalized linear model function *glm ()* with a binomial error distribution and a logit link function. The *p*-values were corrected for multiple testing at a false discovery rate of 5% (Benjamini and Hochberg, 2000). All statistical analyses were performed in the open source software R 3.4.3 (R Core Team, 2017).

### Bud set scoring and data analysis

Bud set was scored in late summer 2017, from the beginning of August to the end of September, of the apical bud of the ramets in the greenhouse. We used a seven stage scoring system to cover onset and duration of bud set developed for *P. nigra* by Rohde et al. (2011). Scores go from 3 (growing apical meristem) to 0 (fully developed bud), in 0.5 intervals (Supplementary Table 5). Observations were performed once a week resulting in 6 dates of observations for in total 812 individual ramets of 65 donor trees identified as Lombardy poplar based on the results of the SSR analysis (Supplementary Table 1). The phenological scores for bud set (Bs) were modeled using cumulative logistic regression using the R package ordinal (Christensen, 2015). Cumulative link mixed models are fitted in this package with the command “*clmm.”* This models the chance (p) to maximally have reached a given level of the ordinal response variable bud set (Bs). The bud set scores were ordered from 3 to 0, so that the chance of maximally reaching e.g., bud set score 1.5 included scores: 3, 2.5, 2, and 1.5. The home site of the donor trees (S) was in the fixed part (categorical variable) of the model. For the ramets, weight data was correlated with total carbon - nitrogen ratio (*r* = −0.504, *p*-value < 2.2e-16), therefore only CN was included as a covariate in the model in the fixed part to correct for transgenerational plasticity of bud set due to nutrient condition. Day (D) was also added in the fixed part to account for the different observation days. The random part (random intercept) consisted of a unique donor tree identity (TID) and a unique identity code for each ramet (RID). The latter accounted for the repeated observations on the same ramets. This resulted in the following cumulative link mixed model:

(1) $$\begin{array}{l} \;\log(\frac{\;P_{Bs}}{1-P_{Bs}})=\;\alpha_{T}-\beta_{D}\times D(fixed)\\ -\beta_{S}\times S(fixed)-\;\beta_{CN}\times CN(fixed)\\ -ranef_{TID}(random)-ranef_{RID}(random) \end{array}$$

*α*_*T*_ is a threshold value indicating the passing on from one level of the ordinal bud set response variable to the next. β_*D*_, *β*_*S*_, and *β*_*CN*_ are the estimated coefficients for the fixed covariates *D, S*, and *CN*. *Ranef*_*TID*_ and *ranef*_*RID*_ are the random effect coefficients for all levels of the variables TID and RID.

The timing of bud set across the different donor trees was assessed by calculating the DOY (day of the year) when the probability for having reached maximally bud set score 1.5 attained 50% (D_50%_). A D_50%_-value for a given donor tree therefore indicated the day that half of the ramets of this tree had reached maximally the given stage of the phenophase, taking into account a mean value for CN (CN = 12).

(2) $$D_{50\%}=\frac{\alpha_{T}-\beta_{CN}\times 12-\beta_{S}-\;ranef_{TID}(random)}{\beta_{D}})$$

The D_50%_ values were used to calculate Pearson correlation coefficients with climate variables from the home sites of the donor trees; the average temperature (°C) of the coldest (January) and warmest (July) month in the year and the average monthly potential evapotranspiration rate (mm month^−1^). We used Welch's test (Welch, 1938; Ruxton, 2006), a *t*-test for unequal variances, to determine the statistical significance between the methylation state of each epilocus and the D_50%_ values. For this analysis, we ignored possible somatic mutations and scored fragment absence as unmethylated (score: “0”). Loci scored as present (“1”) in only one ramet, or in all ramets except one, were discarded.

## Results

### Identification of multilocus genotypes

**Figure 1:**
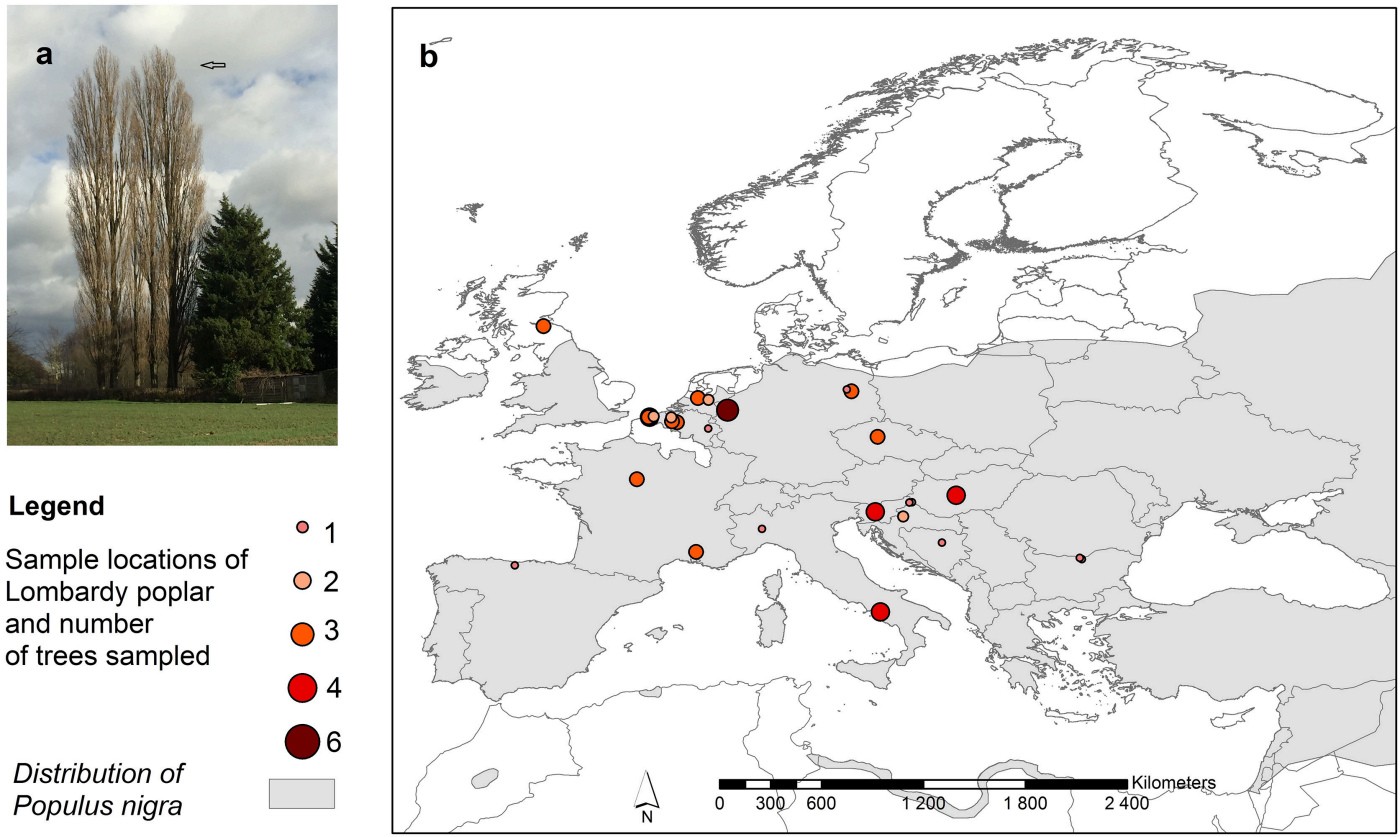
**(a)** Lombardy poplars, **(b)** Distribution of collection sites of Lombardy poplars and number of trees sampled per location. The map also shows the distribution range of the *Populus nigra* L. in Europe, including natural and naturalized stands (EUFORGEN http://www.euforgen.org/species/populus-nigra/).

The 94 sampled putative Lombardy poplar trees analyzed with 11 SSR markers resulted in 15 different multilocus genotypes (MLG) (Supplementary Table 4) of which the most common genotype (G01) was shared by most of the donor trees (65 trees) (Supplementary Table 1) and therefore we consider it to be the genotype of the “true” Lombardy poplar. Furthermore, two multilocus genotypes, representing four (G07) and one (G14) samples, respectively, differed from G01 for only one out of the 22 alleles. They were considered as Lombardy poplars representing the identical genotype but with a possible somatic mutation at locus WPMS05 (mismatch of one repeat), resulting in 72 (75%) individual trees identified as Lombardy poplars sampled in 13 different countries at 37 locations (Figure 1). Other MLGs showed differences with the most common MLG (G01) for in total 7 to 11 alleles. Remarkably, they share alleles with the “true” Lombardy poplar to a high degree. Three of the latter MLGs showed maximum one difference with the most common MLG at each locus and are likely direct sexual offspring, i.e., the result of a cross between a *P. nigra* female and the Lombardy poplar as the paternal parent. The mean genotyping error rate calculated from the 14 replicates was 0.003 (0.3%).

### Variation in DNA methylation

**Figure 2:**
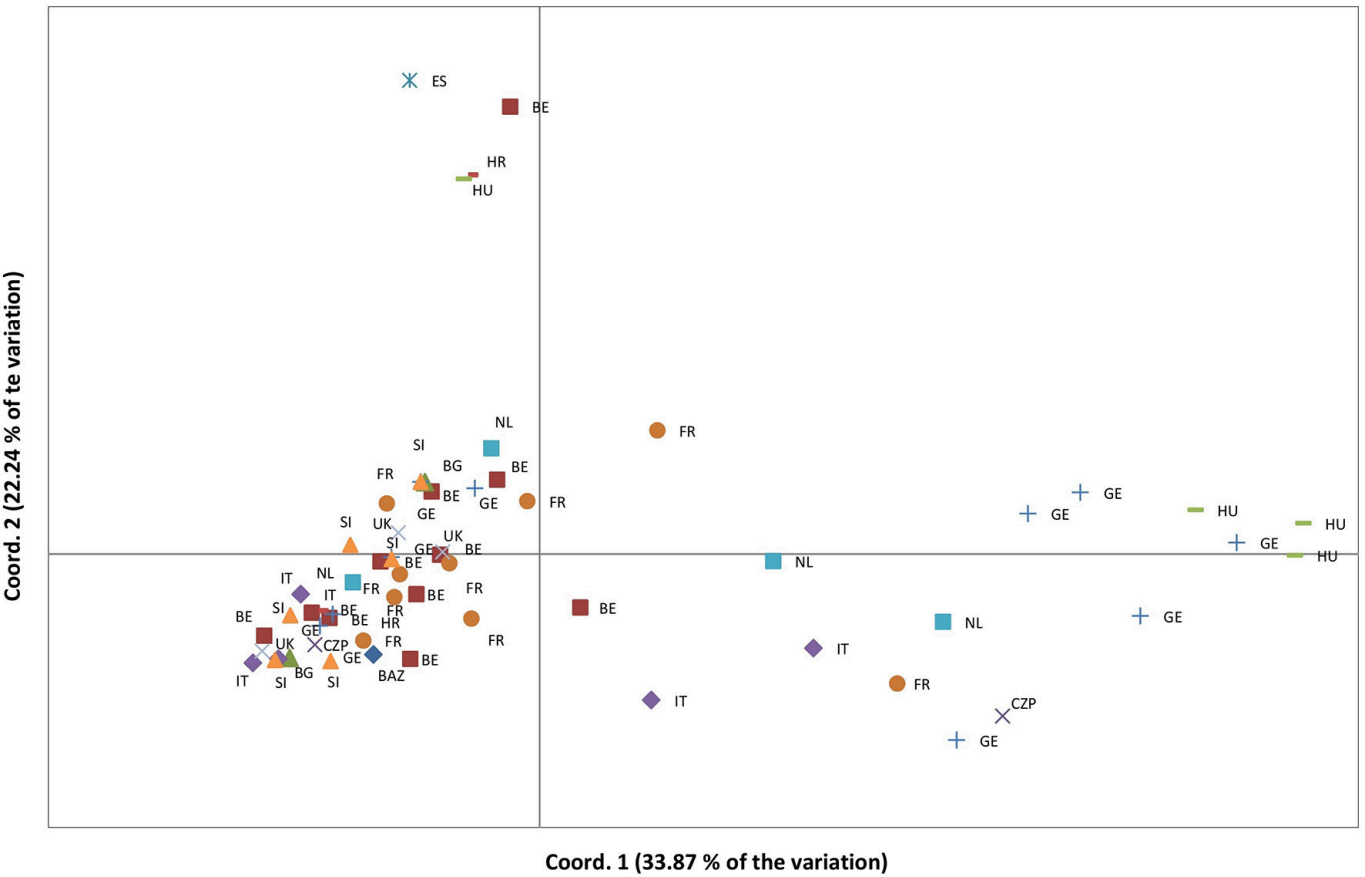
Principal coordinate analyses based on the epigenetic distances calculated on 50 polymorphic MS-AFLP loci and for vegetative offspring grown in a common environment, originating from 60 Lombardy poplar trees collected in 13 countries and representing a single microsatellite genotype. Country codes are explained in Supplementary Table 1.

Complete DNA fragment profiles for both enzyme combinations and the seven primer pairs (7 × 2 = 14 DNA-fragment profiles) were obtained for vegetative offspring of 60 out of 65 (92%) “true” Lombardy poplar donor trees grown in the common environment and collected in 13 countries at 25 different geographic locations with a mean number of trees per location of 2.4 (range: 1 to 6) (Supplementary Table 1). The mean error rate for all primer pairs calculated based on the 14 replicated leaf samples was 2.8% (Table 1), which was within the 2–5% technical error rate range usually found in AFLP studies (Bonin et al., 2004). The transformation of the *Eco*RI/*Hpa*II—*Msp*I fragment profiles into a binary data matrix representing epigenetic differentiations, resulted in 216 epiloci comprising 94 methylation-susceptible epiloci, of which 65 (68%) were polymorphic among the 60 Lombardy poplars (Table 2). The number of variable positions in the epigenetic analysis (65) was significantly higher than the estimated scoring error rate of 2.8% (*t*-test, *p* < 0.00005), indicating significant epigenetic differentiation between samples. In contrast to the SSR genotype, all the 60 epigenotypes were unique. Pairs of epigenotypes differed by at least 2 (3%) and up to 23 (56%) epiloci of the 65 epiloci analyzed [mean pairwise differences: 17 (26%), Supplementary Figure 1]. Fifteen epiloci with missing values, as a result of scoring fragments of both *Hpa*II and *Msp*I cuts considered as uninformative, were removed prior to further analyses. We estimated a Shannon's Diversity Index of 0.386 (SD: 0.163). The differentiation between country of origin of the donor trees is visible in the PCoA; in fact, ramets obtained from Lombardy poplar trees located in Bosnia, Spain, Croatia, and Hungary clustered apart from the rest of the analyzed samples (Figure 2). This was also reflected by the results of the AMOVA analysis. Significant epigenetic differentiation was found between countries [Φ_ST_ = 0.078, *p*_(rand__> =__data)_ = 0.015]. There was no significant correlation between the pairwise distances calculated for MS-AFLP markers and the geographic distances of sampled trees (km) using a Mantel test [*r*^2^ = 0.100, *p*_(rand__> =__data)_ = 0.07].

Fragment absence scored as unmethylated state resulted in 68 polymorphic methylation-susceptible epiloci. We found significant, simple logistic regressions for 11 out of 68 epiloci with a climate variable as explanatory variable (Supplementary Table 6). Several climate variables are correlated with each other (with *r*2 > 0.8: average January and average March temperature, average January temperature and number of frost days, average January temperature and PET, average March temperature and PET, number of frost days and PET). Therefore, for some epiloci multiple significant associations with climate variables were detected. However, after FDR correction none of these models remained significant meaning that none of the epiloci could significantly be correlated with a climate variable.

### Variation in bud set

**Figure 3:**
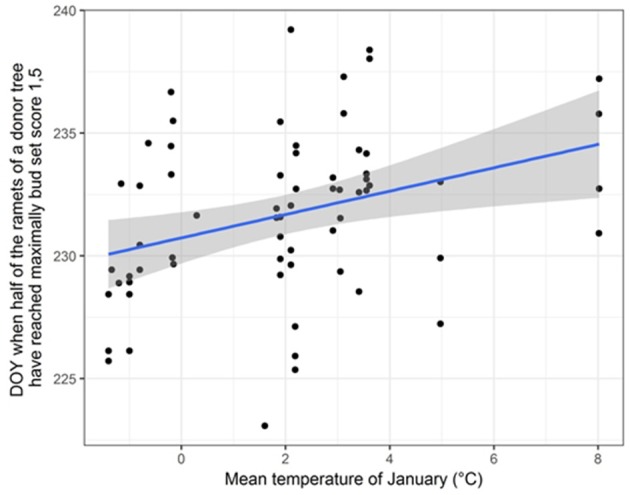
Scatter plot displaying the correlation between the average January temperature at the home-site of the donor trees and the day of the year (DOY) when half of the ramets of a donor tree have reached the probability for having maximally bud set score 1.5 (D_50%_ -values). A linear regression line with 95% confidence intervals is shown.

We found significant differences in bud set between ramets originating from 65 “true” Lombardy poplar donor trees located at different sampling locations, while accounting for physiological effects in terms of the ramets' nutrient condition (Supplementary Table 7). Furthermore, we found significant correlations between timing of bud set and the mean temperature in January (*r* = 0.34; *p*-value = 0.006) (Figure 3) and the average monthly potential evapotranspiration (*r* = 0.33; *p*-value = 0.008), indicating that ramets originating from donor trees at sites with warmer winter temperatures set buds later compared to ramets originating from donor trees at sites with colder winters. No correlation was found with average July temperature (*r* = 0.03; *p*-value = 0.80). Only for one locus (m110; locus amplified with primer combination four and resulting in a fragment of size 186.04 bp), the D_50%_-values were significantly different for the two methylation states (*t* = −2.36, df = 43.25, *p*-value = 0.02). After FDR correction, this relation became non-significant.

## Discussion

### Patterns of DNA methylation variation

In addition to the genetic component, epigenetic variation has been suggested to contribute to the phenotypic plasticity and the adaptive potential of individuals and populations to cope with changing environmental conditions (e.g., Bräutigam et al., 2013; Alsdurf et al., 2016; Whipple and Holeski, 2016). In this context, the Lombardy poplar provides a convenient study system to investigate landscape-level patterns of epigenetic variation along pronounced environmental gradients because it represents a single genotype, it is easy to identify, very widespread and easy to propagate by cuttings. We found high levels of DNA methylation variation [65 (68%) CpG methyl polymorphisms] in asexual reproduced offspring grown in a common greenhouse environment, with a small but significant part of this epigenetic variation (7.8%) distributed among the countries of origin of the donor trees. The observed percentage of polymorphic genome-wide cytosine methylated sites is similar as reported for the genetically depauperate tree species *Pinus pinea* L. (65%) in natural populations covering the entire distribution area of the species in Spain (Sáez-Laguna et al., 2014). Because the Lombardy poplar plants under study represent a single genotype, we assume that the observed methylation variation under the controlled greenhouse conditions in this work is most likely caused by differences in the environment of the donor trees (maternal environment) (Richards et al., 2012; Preite et al., 2015; Whipple and Holeski, 2016). Although microenvironmental variation among plants, differences in the ontological status of the ramets, epigenetic mutations and/or differences in storing time of the ramets, may be associated to some extent with methylation variability. Only a few studies have taken advantage of common garden approaches for studying the persistence of environmental-induced epigenetic variation over generations through clonal reproduction. For instance, in the Japanese knotweed [*Fallopia japonica* (Houtt.) Ronse Decr.], Richards et al. (2012) found evidence of the persistence of naturally induced epigenetic marks through clonal reproduction. Comparing the same individuals, Richards et al. (2012) found nearly five times as many variable positions detected in the epigenetic MS-AFLP analysis compared to the genetic AFLP analysis. Verhoeven et al. (2010), on the other hand, triggered stress-induced epigenetic variation in dandelion (*Taraxacum officinale* F. H. Wigg.) by chemical induction of herbivore and pathogen defenses, and found that the majority of artificially induced epigenetic variation was asexually inherited over the next generation. Our results contribute to the evidence that the environment can have an additional role in generating asexual heritable variation through epigenetic marks. Even so, multi-generation common garden experiments across multiple environments will be necessary to provide insights into the stability of the epigenetic variation found beyond the first generation and in the effect of the environment on the phenotype (Whipple and Holeski, 2016).

### Epigenetic associations with climate variables

Although our results suggest asexual heritable epigenetic variation, we could not directly link DNA methylation variation with relevant climate variables at the landscape scale (>1,000 km). A number of studies have investigated the role of epigenetics in response to environmental conditions in plants at the local or regional scale (e.g., Richards et al., 2012; Medrano et al., 2014; Dubin et al., 2015; Foust et al., 2016), although only a few have studied genome-wide natural DNA methylation variation in relation to climate at the landscape scale (Preite et al., 2015; Gugger et al., 2016; Keller et al., 2016). Using the MS-AFLP technique, significant correlations could be detected between single-nucleotide methylation polymorphisms and the environment at limited spatial scales (scale length <100 km) (e.g., Richards et al., 2012; Medrano et al., 2014; Foust et al., 2016) while, similar to this study, no or very weak correlations are documented on larger spatial scales (Preite et al., 2015; Foust et al., 2016). A potential explanation for these contra-intuitive observations could be that site-specific conditions may cloud the ability to detect significant correlations with spatial or climate variables over larger distances when using a relative small number of MS-AFLP marks (Foust et al., 2016). Using more powerful whole-genome bisulphite-sequencing methodologies, stronger associations were indeed found at the single-nucleotide methylation level with spatial structure and climate variables, especially temperature, in the long-lived *Quercus lobata* Née 1801 (Gugger et al., 2016) and in *A. thaliana* (Dubin et al., 2015; Keller et al., 2016). These studies documented several climate- and space-associated single methylated variants (SMVs). Many of them were CG-SMVs that tended to occur in or near genes involved in plant's response to environment, suggesting that gene body CG-methylation variation may play an important role in plant's response to adapt to variable climatological conditions (Platt et al., 2015; Gugger et al., 2016; Keller et al., 2016). Moreover, in *A. thaliana*, more strong methylation associations with climate were found at the regional scale (Sweden) compared to the broader geographical scale (Eurasia) (Keller et al., 2016). As discussed by Keller et al. (2016), the epigenetic associations with climate variables appear to depend on the geographic scale as well as the sample size. Mechanisms of local adaptation may be restricted geographically such that global models obscure patterns occurring within regions (Lasky et al., 2012; Keller et al., 2016).

### Epigenetic effects on the heritable phenotype

Despite the increasing awareness of the potential role of naturally induced epigenetic changes in an organism's capacity to adapt to its local environment, the contribution of epigenetic effects to the heritable phenotype is largely unexplored (reviewed by Verhoeven et al., 2016; Richards et al., 2017). Here, we found a significant variation in timing of cessation of growth (bud set) in ramets collected from long-lived Lombardy poplars growing over a large geographic range in contrasting environments, after correcting for physiological effects. By growing clonal offspring in a greenhouse experiment, we were able to show that ramets from colder origins set bud slightly quicker than ramets obtained from warmer origins. The average January temperature and the average monthly potential evapotranspiration of the maternal environment were significantly correlated with time of bud set and thus with the timing of the cessation of growth elongation in the common environment. Although we did not study bud set on the donor trees at the collection sites, former studies in *Populus* have shown that trees from more southern locations indeed ceased shoot growth later in summer than trees from more northern origins (e.g., Farmer, 1996; Rohde et al., 2011; Evans et al., 2016). Moreover, the seasonal growth cycle is known to be genetically controlled in *Populus*, and it is considered ecologically important and even subjected to divergent selection (Rohde et al., 2011; Evans et al., 2016). Different environmental conditions can influence the expression levels of specific genes, resulting in reaction norms, i.e., the specific way that different genotypes respond phenotypically to various environmental signals (e.g., Grenier et al., 2016). However, the contribution of phenotypic plasticity to variation in bud set, or the degree to which the expression of genes is modulated by different environmental conditions, is not well-understood (Rohde et al., 2011). Our results suggest that, in Lombardy poplar, epigenetic marks contribute to variation of phenotypic response that can be transferred to the asexually reproduced offspring resulting in locally adapted ecotypes. So far, effects of DNA methylation on the transgenerational phenotype in the absence of genetic diversity, has only been studied on *A. thaliana* using epigenetic recombinant inbred lines (epiRILs). These studies detected heritable phenotypic effects for root length and flowering time (Zhang et al., 2013; Cortijo et al., 2014). In our study, we could not find direct evidence for the link between epiloci and bud set. The complex genetic architecture behind bud set in *Populus* sp. with many genes of small effects (Rohde et al., 2011; Evans et al., 2016), combined with the low local sample size in our study, may explain why we did not find direct evidence for specific epiloci correlated with time of bud set. However, the results suggest that heritable phenotypes also exist under natural conditions and in the absence of genetic variation. It is therefore plausibly that local adaptive variation, as a result of epigenetic variation and driven by the climate, exists in Lombardy poplar. Although our study system, the Lombardy poplar, does not consist of natural populations, it is likely that similar mechanisms occur in natural populations of the European black poplar.

## Conclusion

The results of our study support the prediction that epigenetic-based transgenerational inheritance might be relevant for evolution in rapidly changing environments (e.g., Bräutigam et al., 2013; Gugger et al., 2016; Whipple and Holeski, 2016). Whether such transgenerational effects persist over several years and over multiple clonal generations requires further investigation combining epigenomics with common garden experiments over multiple generations.

## Data availability

The datasets for this study (the binary data matrix of the scored MS-AFLP profiles and the data of the bud set observations) are available on Dryad Digital Repository, doi: 10.5061/dryad.2gf700s.

## Author contributions

AV, BH, SC, and KC designed the study, AV organized the research and collected the data, AV, KC, and KV analyzed the data. AV, KC, and KV contributed to the interpretation of the data. AV, KC, RB, SC, AC, FG, BH, MS, and KV contributed to the writing of the manuscript and all authors approved the final manuscript.

### Conflict of interest statement

The authors declare that the research was conducted in the absence of any commercial or financial relationships that could be construed as a potential conflict of interest.
