# Supplementary material for: Variability in DNA Methylation and Generational Plasticity in the Lombardy Poplar, a Single Genotype Worldwide Distributed Since the Eighteenth Century
Source: Front Plant Sci. 2018 Nov 13;9:1635. doi: 10.3389/fpls.2018.01635 (PMC6242946; doi:10.3389/fpls.2018.01635)
Supplement: Supplementary file 2 [file Table_2.DOCX]

Supplementary Material

Epigenetic variation and generational plasticity in the Lombardy poplar, a single genotype worldwide distributed since the 18^th^ century

An Vanden Broeck*, Karen Cox, Rein Brys, Stefano Castiglione, Angela Cicatelli, Francesco Guarino, Berthold Heinze, Marijke Steenackers, Kristine Vander Mijnsbrugge

*** Correspondence:** Corresponding Author: [an.vandenbroeck@inbo.be](mailto:an.vandenbroeck@inbo.be)

Supplementary Table 2. List of the microsatellite markers used for the identification of *Populus nigra* genotypes.

| **Left Primer /forward (5′→3′)** | **Right Primer/reverse (5′→3′)** | **Motif** | **Expected Product Length (bp)** | **Ta (°C)** | **Reference** |
| --- | --- | --- | --- | --- | --- |
| TTCAGAATGTGCATGATGG | GTGATGATCTCACCGTTTG | CTT | 179 - 227 | 52 | 1 |
| TTCTTTTTCAACTGCCTAACTT | TGATCCAATAACAGACAGAACA | GT | 263 - 291 | 52 | 2 |
| CAATCGAAGGTAAGGTTAGTG | CGTTGGACATAGATCACACG | GA | 198 - 220 | 52 | 1 |
| CTCGTACTATTTCCGATGATGACC | AGATTATTAGGTGGGCCAAGGACT | GTC | 128 - 167 | 52 | 3 |
| GTGGGGATCAATCCAAAAGA | CCCATATCAAACCATTTGAAAAA | CCT | 189 - 201 | 57 | 1 |
| GTGCGCACATCTATGACTATCG | ATCTTGTAATTCTCCGGGCATCT | TTCTGG | 224 - 242 | 57 | 3 |
| AAGAAGAACTCGAAGATGAAGAACT | ACTGACAAAACCCCTAATCTAACAA | TGG | 207 - 228 | 57 | 1 |
| ATTTGATGCCTCTTCCTTCCAGT | TATTTTCATTTTCCCTTTGCTTT | (CT)5AT(CT) | 230 - 250 | 57 | 1 |
| CAGCCGCAGCCACTGAGAAATC | GCCTGCTGAGAAGACTGCCTTGAC | CGT | 221 - 304 | 57 | 3 |
| CAACAAACCATCAATGAAGAAGAC | AGAGGGTGTTGGGGGTGACTA | CCT | 188 - 203 | 57 | 3 |
| AGCCACAGCAAATTCAGATGATGC | CCTGCTGAGAAGACTGCCTTGACA | CAG | 174 - 252 | 57 | 3 |

References: 1; <http://web.ornl.gov/sci/ipgc/ssr_resource.htm>, 2; van der Schoot *et al.* 2000, 3; Smulders *et al.* 2001
