# Supplementary material for: Variability in DNA Methylation and Generational Plasticity in the Lombardy Poplar, a Single Genotype Worldwide Distributed Since the Eighteenth Century
Source: Front Plant Sci. 2018 Nov 13;9:1635. doi: 10.3389/fpls.2018.01635 (PMC6242946; doi:10.3389/fpls.2018.01635)
Supplement: Supplementary file 3 [file Table_3.DOCX]

Supplementary Material

Epigenetic variation and generational plasticity in the Lombardy poplar, a single genotype worldwide distributed since the 18^th^ century

An Vanden Broeck*, Karen Cox, Rein Brys, Stefano Castiglione, Angela Cicatelli, Francesco Guarino, Berthold Heinze, Marijke Steenackers, Kristine Vander Mijnsbrugge

*** Correspondence:** Corresponding Author: [an.vandenbroeck@inbo.be](mailto:an.vandenbroeck@inbo.be)

Supplementary Table 3. Scoring parameters used in the automatic scoring program RawGeno

| **scoring parameters** | **Primer combination** | | | | | | |
| --- | --- | --- | --- | --- | --- | --- | --- |
|  | 1 | 2 | 3 | 4 | 5 | 6 | 7 |
| maximum bin width | 1.2 | 1.5 | 1.2 | 1.5 | 1.1 | 1.3 | 1.5 |
| minimum bin width | 1 | 1 | 1 | 1 | 1 | 1 | 1 |
| minimum peak heights (rfu) | 250 | 200 | 150 | 250 | 125 | 180 | 250 |
