# Supplementary material for: Variability in DNA Methylation and Generational Plasticity in the Lombardy Poplar, a Single Genotype Worldwide Distributed Since the Eighteenth Century
Source: Front Plant Sci. 2018 Nov 13;9:1635. doi: 10.3389/fpls.2018.01635 (PMC6242946; doi:10.3389/fpls.2018.01635)
Supplement: Supplementary file 5 [file Table_5.DOCX]

Supplementary Material

Epigenetic variation and generational plasticity in the Lombardy poplar, a single genotype worldwide distributed since the 18^th^ century

An Vanden Broeck*, Karen Cox, Rein Brys, Stefano Castiglione, Angela Cicatelli, Francesco Guarino, Berthold Heinze, Marijke Steenackers, Kristine Vander Mijnsbrugge

*** Correspondence:** Corresponding Author: [an.vandenbroeck@inbo.be](mailto:an.vandenbroeck@inbo.be)

Supplementary Table 5. Scoring scheme for bud set in poplar

| Stage | Description |
| --- | --- |
| 3 | Apical shoot fully growing |
| 2.5 | Internode elongation ceased |
| 2 | Internode elongation ceased, no bud visible |
| 1.5 | Transition to bud structure |
| 1 | Apical bud visible, bud still open |
| 0.5 | Closed apical bud |
| 0 | Bud set |
