# Supplementary material for: Variability in DNA Methylation and Generational Plasticity in the Lombardy Poplar, a Single Genotype Worldwide Distributed Since the Eighteenth Century
Source: Front Plant Sci. 2018 Nov 13;9:1635. doi: 10.3389/fpls.2018.01635 (PMC6242946; doi:10.3389/fpls.2018.01635)
Supplement: Supplementary file 6 [file Table_6.DOCX]

Supplementary Material

Epigenetic variation and generational plasticity in the Lombardy poplar, a single genotype worldwide distributed since the 18^th^ century

An Vanden Broeck*, Karen Cox, Rein Brys, Stefano Castiglione, Angela Cicatelli, Francesco Guarino, Berthold Heinze, Marijke Steenackers, Kristine Vander Mijnsbrugge

*** Correspondence:** Corresponding Author: [an.vandenbroeck@inbo.be](mailto:an.vandenbroeck@inbo.be)

Supplementary Table 6. Results of the simple logistic regression analyses with a significant predictor variable (without FDR correction).

SE; standard error, JAN; the average temperature of January, MAR; the average temperature of March, JUL; the average temperature of July, PRATE; the average monthly precipitation rate, FRST; the average number of frost days per year, PET; the average monthly potential evapotranspiration rate, CN; the total carbon – total nitrogen ratio.

*, P < 0.5; **, P < 0.01, ***, P < 0.001

| Epilocus | Estim ate intercept (SE) | Variable | Estimate variable (SE) |
| --- | --- | --- | --- |
| m26 | 1.60 (1.93) | **PRATE** | -0.08 (0.04)* |
| m30 | -4.51 (1.26)*** | **JAN** | 0.46 (0.23)* |
| m30 | -7.90 (2.81)** | MAR | 0.68 (0.33) * |
| m30 | -8.86 (3.25)** | **PET** | 0.18 (0.09)* |
| m83 | 1.66 (1.57) | **PRATE** | -0.07 (0.03)* |
| m112 | 13.27 (5.42)* | **JUL** | -0.54 (0.25)* |
| m131 | -8.07 (3.09)** | CN | 0.53 (0.23)* |
| m152 | 1.66 (1.30) | **PRATE** | -0.06 (0.02)* |
| m171 | 1.51 (1.05) | **PRATE** | -0.04 (0.02)* |
| m179 | -8.23 (3.35)* | JUL | 0.33 (0.16)* |
| m184 | -3.36 (0.80)*** | JAN | 0.39 (0.17)* |
| m184 | -5.58 (1.80)** | MARC | 0.53 (0.23)* |
| m184 | -7.66 (2.39) | PET | 0.17 (0.07)* |
| m185 | 2.26 (0.54)*** | JAN | -0.30 (0.13)* |
| m185 | 4.92 (1.42)*** | MARC | -0.51 (0.20)** |
| m185 | 9.51 (3.28)** | JUL | -0.41 (0.16)* |
| m185 | 5.81 (1.90)** | PET | -0.14 (0.06)* |
| m205 | -3.17 (2.55) | MARC | 0.96 (0.48)* |
| m205 | 7.83 (2.95) ** | FRST | -0.27 (0.12)* |
| m205 | -5.41 (3.36) | CN | 0.64 (0.30)* |
