# Supplementary material for: Variability in DNA Methylation and Generational Plasticity in the Lombardy Poplar, a Single Genotype Worldwide Distributed Since the Eighteenth Century
Source: Front Plant Sci. 2018 Nov 13;9:1635. doi: 10.3389/fpls.2018.01635 (PMC6242946; doi:10.3389/fpls.2018.01635)
Supplement: Supplementary file 7 [file Table_7.DOCX]

Supplementary Material

Epigenetic variation and generational plasticity in the Lombardy poplar, a single genotype worldwide distributed since the 18^th^ century

An Vanden Broeck*, Karen Cox, Rein Brys, Stefano Castiglione, Angela Cicatelli, Francesco Guarino, Berthold Heinze, Marijke Steenackers, Kristine Vander Mijnsbrugge

*** Correspondence:** Corresponding Author: [an.vandenbroeck@inbo.be](mailto:an.vandenbroeck@inbo.be)

Supplementary Table 7. Model statistics for the response variable bud set.

Adinkerke (Belgium) is the standard level of the variable site to which the other homesites of the donor trees are compared to. D; day of observation, CN; the total carbon – total nitrogen ratio, SE; standard error.

| covariate | Estimate | SE | *z* value | *p* value |
| --- | --- | --- | --- | --- |
| D | 0,41 | 0,01 | 47,80 | <0,001*** |
| Avignon | 0,73 | 0,78 | 0,94 | 0,349 |
| Aywalle | 3,61 | 1,10 | 3,29 | 0,001** |
| Berlin | 0,37 | 0,85 | 0,44 | 0,662 |
| Dalkeith | -0,20 | 0,77 | -0,26 | 0,793 |
| Duisburg | 0,05 | 0,67 | 0,08 | 0,939 |
| Elstal | -1,14 | 1,10 | -1,03 | 0,301 |
| Geraardsbergen | 0,49 | 0,79 | 0,62 | 0,535 |
| Ghyvelde | -0,61 | 0,81 | -0,76 | 0,450 |
| Lendava | 0,99 | 1,09 | 0,90 | 0,366 |
| Ljubljana | -0,12 | 0,74 | -0,16 | 0,873 |
| Loiret | 0,26 | 0,79 | 0,33 | 0,741 |
| Nagyvenyim | 1,50 | 0,75 | 2,01 | 0,044* |
| Nieuwpoort | -1,94 | 0,91 | -2,13 | 0,033* |
| Orlovscek | 1,21 | 1,09 | 1,11 | 0,267 |
| Piedmont | -3,04 | 1,08 | -2,81 | 0,005** |
| Prague | 2,09 | 0,79 | 2,66 | 0,008** |
| Salermo | -0,97 | 0,78 | -1,25 | 0,212 |
| Svishtov | -0,46 | 1,08 | -0,42 | 0,673 |
| Utrecht | -0,82 | 0,79 | -1,04 | 0,301 |
| Wageningen | 0,04 | 0,98 | 0,05 | 0,964 |
| Zagreb | -1,55 | 0,94 | -1,64 | 0,101 |
| Žepče | 0,08 | 1,11 | 0,07 | 0,946 |
| Zuydcote | -1,90 | 0,80 | -2,36 | 0,018* |
| Zwalm | 2,34 | 0,78 | 2,98 | 0,003** |
| CN | 0,63 | 0,13 | 4,95 | <0,001*** |
