# Supplementary figures and images for: Variability in DNA Methylation and Generational Plasticity in the Lombardy Poplar, a Single Genotype Worldwide Distributed Since the Eighteenth Century
Source: Front Plant Sci. 2018 Nov 13;9:1635. doi: 10.3389/fpls.2018.01635 (PMC6242946; doi:10.3389/fpls.2018.01635)

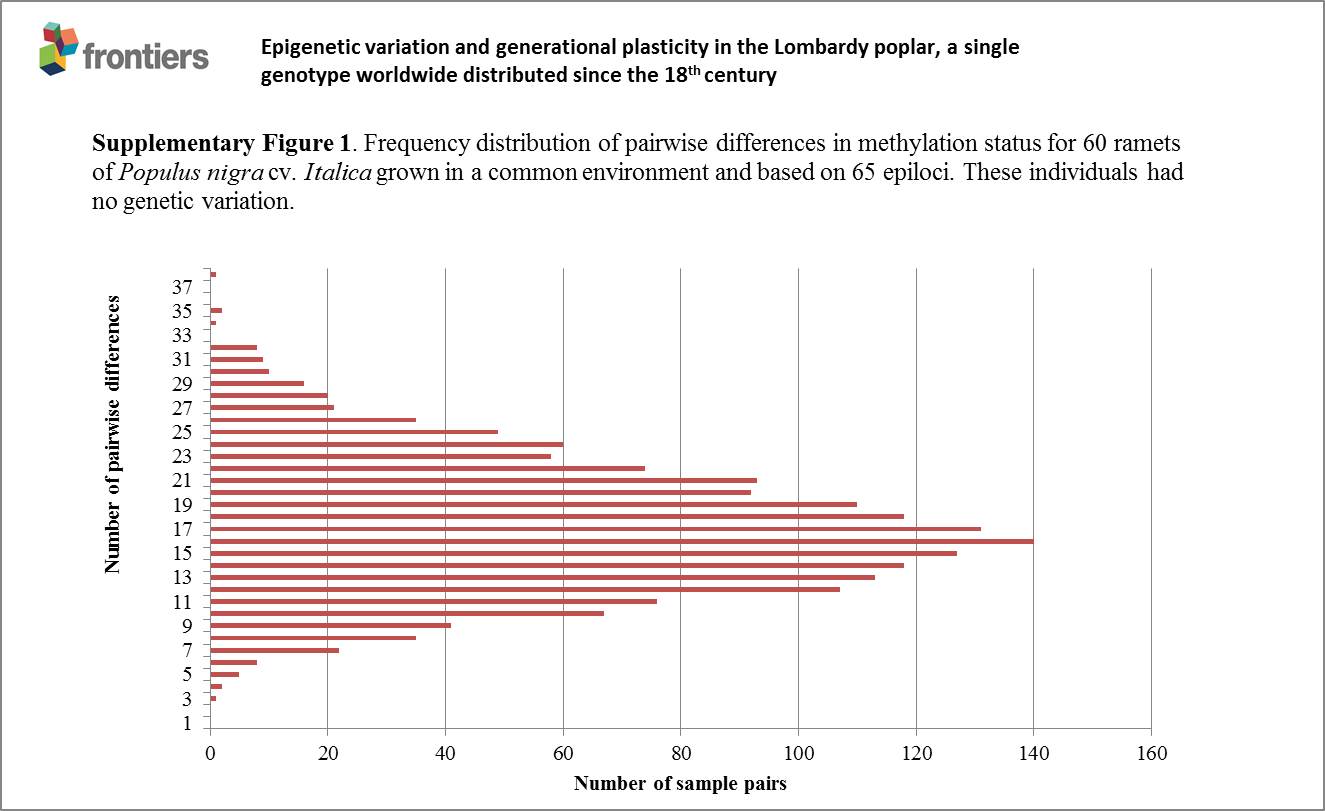

Supplement: Supplementary file 8 [file Image_1.JPEG]
